# 737. Timing, Microbiology, Antibiotic Use, and Financial Impact of Left Ventricular Assist Device Infections: A Prospective Case-Control Approach

**DOI:** 10.1093/ofid/ofad500.798

**Published:** 2023-11-27

**Authors:** Matthew Ficinski, Shannon Glassman, Katrina J Wojciechowski, Jennifer West, Scott Feitell, Emil P Lesho

**Affiliations:** Rochester Regional Health, Rochester, New York; Rochester Regional Health, Rochester, New York; Rochester Regional Health, Rochester, New York; Rochester Regional Health, Rochester, New York; Rochester Regional Health, Rochester, New York; Rochester Regional Health, Rochester, New York

## Abstract

**Background:**

Left-ventricular assist devices (LVAD) are increasingly used to treat advanced heart failure, and infection is a leading complication. Although the main registries such as INTERMACS publish annual quality reports, recently they have called for more details regarding antibiotic exposure and microbiology, which are not typically included in those reports.

**Figure:**
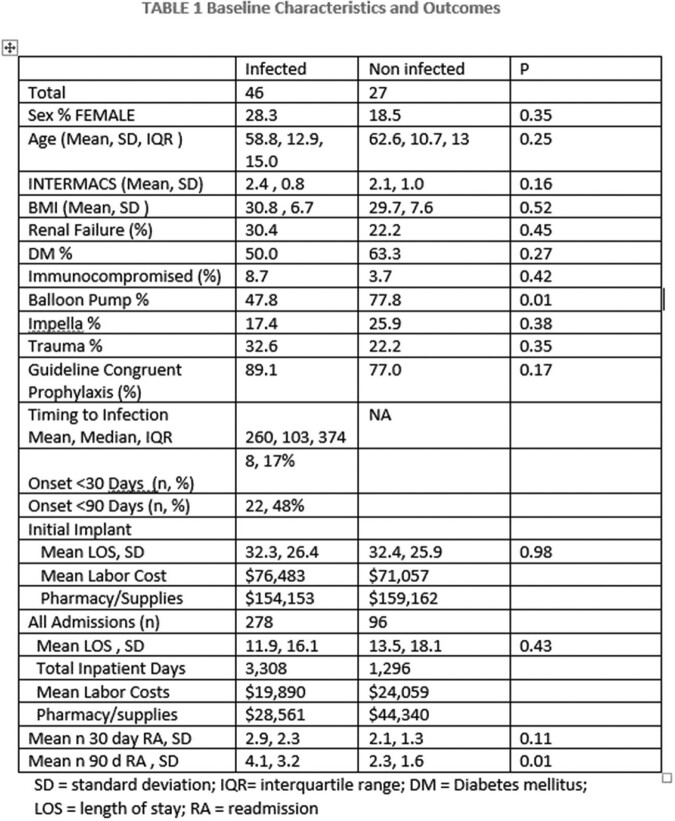

Infection Timing and Antibiotic Usage

**Figure:**
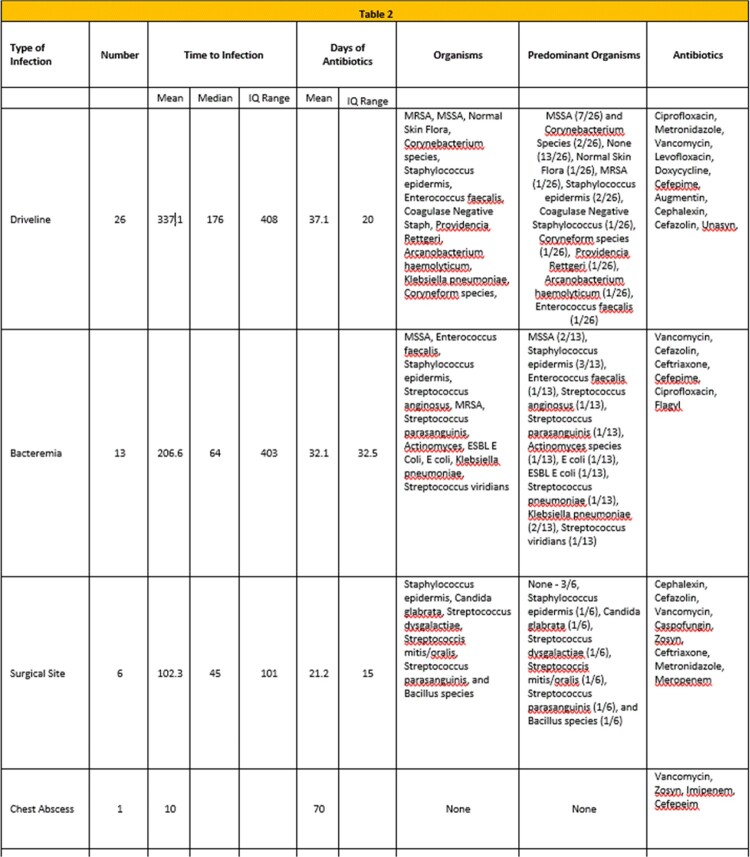

**Methods:**

All patients who receive an LVAD are prospectively monitored by a team of heart failure and infectious diseases specialists until death or they permanently leave the catchment area. All patients from program inception (11-01-18) were included. A cut-off date of 12-3-22 was used to allow for at least of 6-month follow-up period from the last insertion. Internationally standardized infection definitions, a case-control approach, and T and Chi Squared tests were used.

**Results:**

46 of 73 patients developed an LVAD-associated or specific infection. Of those, 57%, 28%, and 13% were driveline, bacteremia, or surgical site infections, respectively. No baseline patient characteristic, including immune status, BMI, co-morbidity, guideline congruent perioperative prophylaxis, or device trauma was associated with infection (Table 1). 48% of infections occurred < 90 days, but there was no association with surgeon, surgical team, or operating room. The mean time to infection was 260 days (IQR 374). The mean days of antibiotic therapy ranged from 21 days for SSI to 37 days for driveline infection (Table 2). *Staphylococcus aureus and Streptococcus* species, and vancomycin and cefazolin were the predominant organisms and antibiotics used, respectively. The mean lengths of stay and costs were similar between infected and uninfected patients, but infected patients required 3x the number of re-admissions and 2x number of hospital days (Table 1).

**Conclusion:**

Most infections occurred after 90 days, with 34% occurring ≥ 1 year after discharge, suggesting a potential role of community or socioeconomic factors. Other confounders that occurred during this study included COVID-19- related issues, staffing shortages, and shortages of chlorhexidine containing skin preparations and other supplies. 59% of patients with an initial infection had recurrent infections. Going forward, we aim to provide similar details for each subsequent infection.

**Disclosures:**

**Katrina J. Wojciechowski, MS, PA-C**, Abbott: Honoraria **Scott Feitell, DO, FACC, FHFSA**, Abbott Laboratories: Advisor/Consultant|Abbott Laboratories: Honoraria

